# Enhanced coalbed methane well production prediction framework utilizing the CNN-BL-MHA approach

**DOI:** 10.1038/s41598-024-65606-z

**Published:** 2024-06-26

**Authors:** Xianxian Li, Xijian Li, Honggao Xie, Cong Feng, Junjie Cai, Yuhuan He

**Affiliations:** 1 College of Mining, Guizhou University, Guiyang, 550025 China; 2 Guizhou Engineering Center for Safe Mining Technology, Guiyang, 550025 China; 3 College of Resource and Environmental Engineering, Guizhou University, Guiyang, 550025 China

**Keywords:** Coalbed methane, Deep learning, Production forecasts, CNN-BL-MHA model, Energy science and technology, Engineering

## Abstract

As the mechanization of the CBM extraction process advances and geological conditions continuously evolve, the production data from CBM wells is deviating increasingly from linearity, thereby presenting a significant challenge in accurately predicting future gas production from these wells. When it comes to predicting the production of CBM, a single deep-learning model can face several drawbacks such as overfitting, gradient explosion, and gradient disappearance. These issues can ultimately result in insufficient prediction accuracy, making it important to carefully consider the limitations of any given model. It’s impressive to see how advanced technology can enhance the prediction accuracy of CBM. In this paper, the use of a CNN model to extract features from CBM well data and combine it with Bi-LSTM and a Multi-Head Attention mechanism to construct a production prediction model for CBM wells—the CNN-BL-MHA model—is fascinating. It is even more exciting that predictions of gas production for experimental wells can be conducted using production data from Wells W1 and W2 as the model’s database. We compared and analyzed the prediction results obtained from the CNN-BL-MHA model we constructed with those from single models like ARIMA, LSTM, MLP, and GRU. The results show that the CNN-BL-MHA model proposed in the study has shown promising results in improving the accuracy of gas production prediction for CBM wells. It’s also impressive that this model demonstrated super stability, which is essential for reliable predictions. Compared to the single deep learning model used in this study, its prediction accuracy can be improved up to 35%, and the prediction results match the actual yield data with lower error.

## Introduction

Coalbed methane (CBM), buried deep within coal seams, serves as a precious energy source that embodies the future of clean energy and facilitates the attainment of the global dual-carbon goal^1,2^. Its distinctive storage mechanism and extraction techniques exhibit humanity’s proficient harnessing of natural resources. China possesses substantial reserves of coalbed methane; nevertheless, there is considerable scope for enhancing its utilization rate^3–5^. The expansive application of CBM in power generation, the chemical industry, and diverse other sectors harbors vast potential, promising to bolster energy supply stability and reduce our reliance on fossil fuels^6–8^. However, accurate forecasting of CBM output confronts numerous challenges, primarily due to intricate geological conditions and complex CBM transport mechanisms^9–11^. Presently, methodologies for anticipating gas production in CBM wells predominantly encompass approaches rooted in mathematical statistics, numerical simulation techniques, and artificial intelligence algorithms^12,13^.

Typical methods for predicting CBM production can be broadly categorized into two approaches: physics-based and mathematical statistics-based^14–16^. The physics-based approach primarily relies on numerical simulation utilizing Darcy’s law^17^. This method necessitates the construction of detailed reservoir models that incorporate intricate geological characteristics and fluid flow dynamics, alongside the careful selection of relevant parameters^15,16^. Nonetheless, its applicability becomes limited in the context of Guizhou’s highly variable coal reservoirs due to their complexity. On the other hand, the mathematical statistics-based methods, specifically including multiple linear regression and support vector regression, facilitate predictions by aligning field data with trend lines^15,18–20^. These methods demand less extensive data and operate with simpler models.

In recent years, AI technologies have revolutionized various industries, with their impact being particularly notable in the field of yield prediction within the oil and gas sector. These technologies have moved beyond traditional modeling paradigms, aligning more closely with actual production scenarios and their unique characteristics^21,22^. Specifically, cutting-edge algorithms like Support Vector Machines, Naive Bayes, and Long Short-Term Memory Networks (LSTMs) leverage their exceptional data processing and analytical abilities to efficiently sift through vast datasets, pinpointing key influencers and constructing sophisticated nonlinear models for precise yield forecasts^14,23,24^. A notable example is the work of Chao Min, Fei Ren, and their team. They not only harnessed machine learning models to accurately predict the output of CBM wells following hydraulic fracturing but also innovatively amalgamated causal discovery theory^25,26^. This methodical approach delved deep into the complex nonlinear interplay of factors affecting production and successfully identified the key drivers of coalbed methane production^25^. This pioneering work not only establishes a robust foundation for production prediction but also paves the way for the development of more holistic reservoir simulation models^26^. Furthermore, researchers including Ruijie Huang have also developed an LSTM neural network model that incorporates the steam injection effect for predicting the production performance of carbonate reservoirs^27^. Compared to traditional numerical simulation methods, this neural network model demonstrates superior overall performance^27^.

Currently, deep learning algorithms have penetrated the research field of coalbed methane production prediction. Under ideal conditions, deep learning models can independently achieve high-precision prediction results^28–30^. However, as the amount of sequential data continues to increase, these models may encounter challenges such as overfitting and gradient explosion. Additionally, the constant changes in geological conditions make it difficult for a single deep learning model to meet the growing demand for prediction^22,31^. Therefore, production prediction models need to have stronger flexibility and adaptability. Ensemble models can more effectively respond to these changes and enhance the robustness of predictions by synthesizing the prediction results of various sub-models. Past research has shown that hybrid models excel in predicting reservoir parameters, oil and gas reservoir permeability, and production^13,32^. In shale gas production prediction, scholars like Wangqiang have utilized the linear model in the hybrid prediction technique (MNGM-ARIMA) to correct nonlinear prediction results. This strategy cleverly combines the advantages of both linear and nonlinear models, achieving remarkable prediction results^33^. In the field of oil reservoir production prediction, Yuan Zhenyu and his team successfully constructed the HDNN model by integrating Convolutional Neural Networks (CNN) and Multi-Layer Perceptrons (MLP) to address prediction problems involving multiple sources and mixed types, accurately predicting production in oil development zones^34^.

This study first employs a one-dimensional convolutional neural network (1D CNN) to perform deep feature extraction on the data, aiming to capture critical information, reduce redundant computations, and enhance data accuracy. Subsequently, the extracted features are passed to the BiLSTM layer for temporal modeling, thereby capturing the dependencies within the time series. To further enhance the expressive power of the features, we introduce a multi-head attention layer, which can fine-tune the weight of each feature through parallel processing of multiple attention heads. This model not only captures the key dynamic parameters of coalbed methane from a bidirectional perspective but also utilizes the multi-head attention mechanism to achieve efficient parallel feature weighting and output computation. The proposed CNN-Bi-LSTM model combined with a multi-head attention mechanism (CNN-BL-MHA model) exhibits excellent flexibility and adaptability in predicting coalbed methane well production. It can accommodate various datasets and task requirements, potentially providing solid technical support for the coalbed methane extraction industry.

## Methodology

In recent years, deep learning algorithms have demonstrated their capability in handling large-scale data for time-series prediction, effectively learning feature representations without the need for manual feature design. However, a single deep learning model applied to different sequences of data often exhibits various limitations, including overfitting and gradient explosion, among others^35^. Based on the aforementioned considerations, this paper employs a CNN for extracting features from experimental data, a Bi-LSTM for time-series prediction, and introduces a multi-head attention mechanism as a scoring function to compute the attention weights of each position relative to other positions. The objective is to improve the prediction performance of the coalbed methane well model and establish a solid theoretical basis for the exploration and development of coalbed methane wells.

### CNN feature extraction

Convolutional Neural Network (CNN) is a deep learning model commonly used for feature extraction. The CNN model mainly consists of convolutional layers, pooling layers, and fully connected layers. Among them, the convolutional layer is the core component of the CNN^24,36^. It performs feature extraction and feature mapping on input data through multiple convolution kernels. The pooling layer reduces data dimensionality and computational complexity through downsampling^37^. The fully connected layer processes the output results from the convolutional and pooling layers, ultimately outputting classification results.

**Figure 1:**
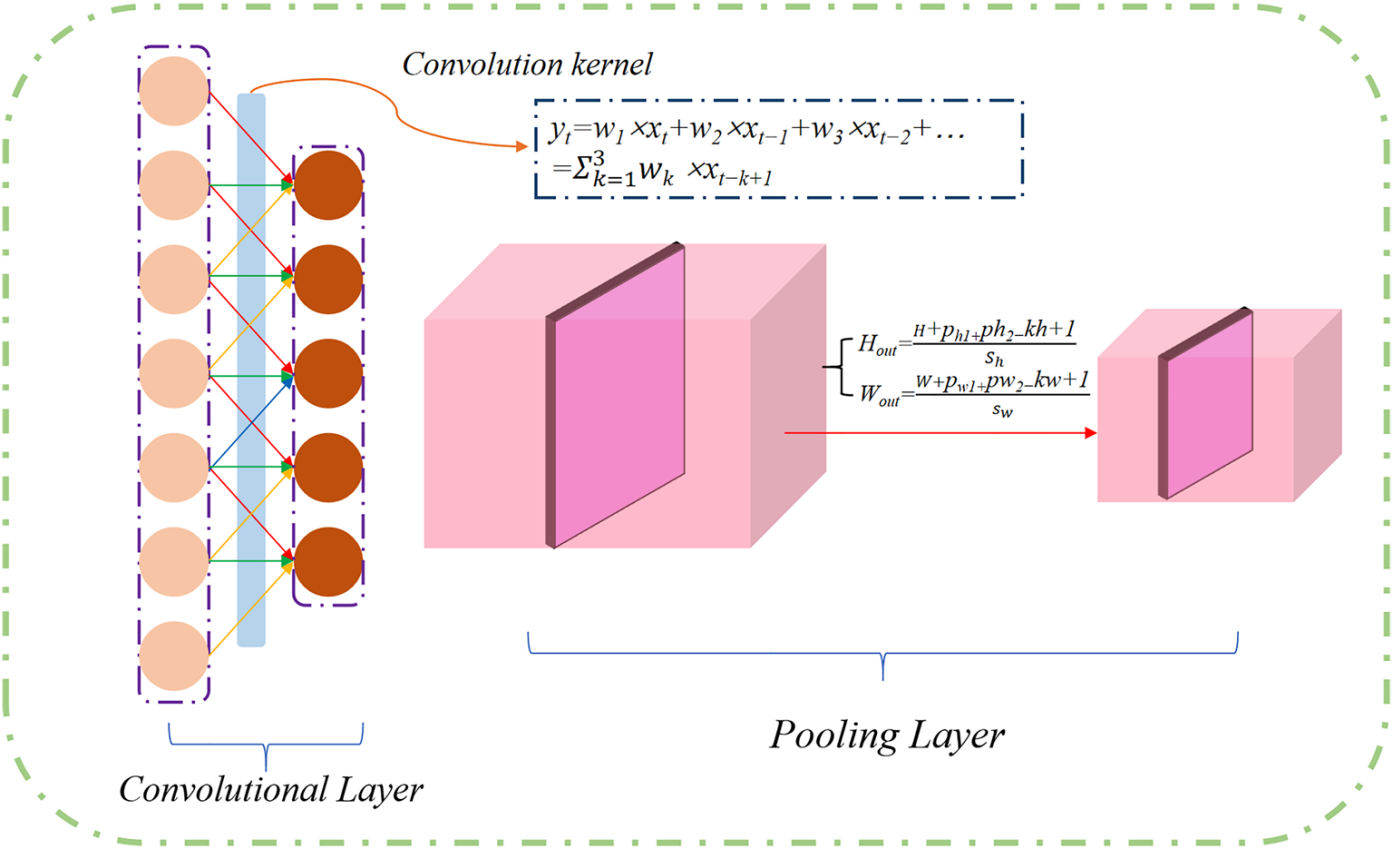
Main structure of convolutional neural network.

The feature extraction process of CNN mainly relies on the synergistic effect of convolutional layers and pooling layers^37^. In the convolutional layer, convolution kernels generate feature maps by sliding over the input data and performing convolution operations, effectively extracting local feature information from the input data^38^. Subsequently, these feature maps undergo a nonlinear transformation through an activation function layer, enhancing their feature representation capabilities. Following this, the pooling layer performs dimensionality reduction on these nonlinearly transformed feature maps, further refining the key features^38^. This process is illustrated in Fig. 1.

For sequential data, CNN models typically employ one-dimensional convolution operations in the convolutional layer, utilizing filters to perform convolution on the input data, capturing local features of the time series, and enhancing nonlinear expression capabilities through activation functions^39^. The pooling layer further compresses data dimensionality, emphasizing key features. The formula is as follows^38^:

1 $$y_{t} = \sum\limits_{i = 0}^{K - 1} {w_{i} \cdot x_{t - 1} + b}$$

2 $$f(x) = \max (0,x)$$

3 $$y_{i} = \frac{1}{N}\sum\nolimits_{j = 1}^{N} {x_{ij} }$$

where $y_{t}$ is the output of the time step (t), $w$ represents the weights, b represents the bias, $f(x)$ is the ReLU activation function.

### Bi-LSTM

Compared to traditional machine learning methods, LSTM has stronger nonlinear learning capabilities to store sequential data. However, LSTM can only be trained and propagated in a unidirectional manner, from left to right. In practical sequence prediction, it is necessary to consider both forward and backward inputs simultaneously to improve the model’s accuracy, The Bi-LSTM model can alleviate this limitation^40,41^. The main differences between Bi-LSTM and LSTM lie in the directionality of information flow and the ability to capture patterns in data sequences^42^.

**Figure 2:**
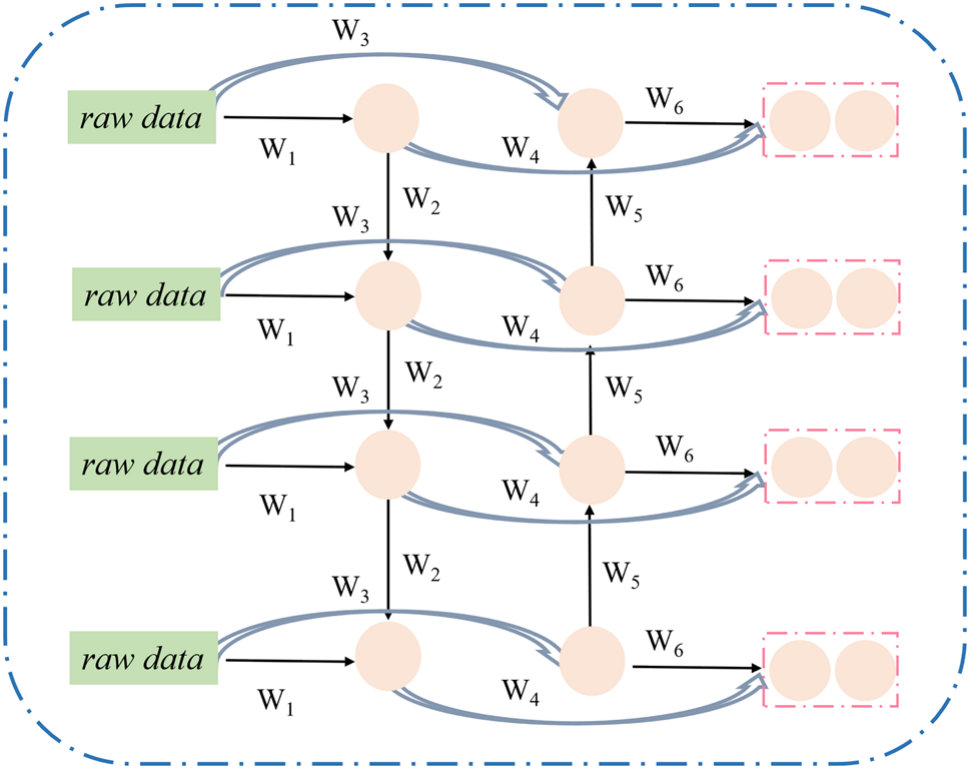
Structure of the Bi-LSTM model.

Bi-LSTM consists of two LSTMs, as illustrated in Fig. 2. The output layer comprises six weights, labeled W1 to W6, connecting to the output layer from both the forward and backward directions^43^. Specifically, W1 and W3 handle inputs for the forward and backward hidden layers, respectively, while W2 and W5 manage information flow within these hidden layers. Meanwhile, W4 and W6 facilitate information transfer from the forward and backward hidden layers to the output layer. To maintain data acyclicity, no information is exchanged between the forward and backward hidden layers. In the forward layer, calculations progress from time 1 to time t, recording the output of the forward hidden layer at each point. Similarly, in the backward layer, we trace back from time t to time 1, documenting the output of the backward hidden layer at each step^44^. The final result is obtained by fusing the outputs from both the forward and backward layers at each time point. The formula for this fusion is as follows^45^:

Forward pass

4 $$h_{t} = f(w_{1} x_{t} + w_{2} h_{t - 1} )$$

Backward pass

5 $$h^{\prime}_{t} = f(w_{3} x_{t} + w{}_{5}h^{\prime }_{t - 1} )$$

6 $$O_{t} = g(w_{4} h_{t} + w_{6} h_{t}^{\prime } )$$

where $\text{W}$ represents weights, $h_{t}$ represents the forward LSTM output vector, $h_{t}^{\prime }$ represents the backward LSTM output vector; $O_{t}$ represents the backward LSTM output vector.

### Multi-head attention mechanism

**Figure 3:**
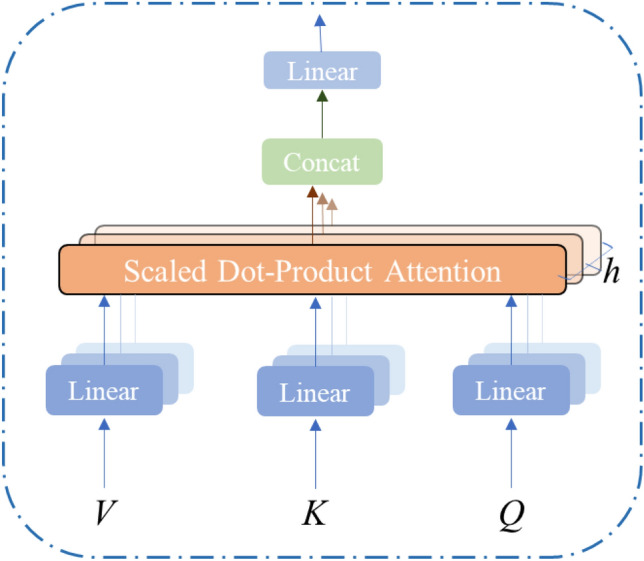
Structure of the multi-head attention mechanism model.

The multi-head attention mechanism serves as a crucial component of the Transformer model, enabling the capture of dependencies across various positions within the input sequence^46^. As shown in Fig. 3, The multi-head attention mechanism achieves this goal by mapping the input sequence to a query (Q), key (K), and value (V) vector, respectively. Subsequently, it employs an attention-scoring function to compute the attentional weights of each position relative to the other positions, effectively capturing dependencies across the input sequence^47,48^. Finally, the output of each attention head is spliced together and then passed through a linear layer to obtain the final output..

The principle of multi-head attention is illustrated in Eqs. (7) and (8):

7 $$Multiheaded(Q,K,V) = Concat(head_{1} , \ldots ,head_{h} )W^{o}$$

8 $$Where head_{i} = Attention\left( {QW_{i}^{Q} ,\;KW_{i}^{K} ,\;VW_{i}^{V} } \right)$$

where, $$W_{i}^{Q} ,\;W_{i}^{K} ,\;W_{i}^{V} \in R^{dmodel \times dk} ;\;W^{O} \in^{Rhdv \times dmodel}$$.

### CNN-BL-MHA gas production prediction modeling

**Figure 4:**
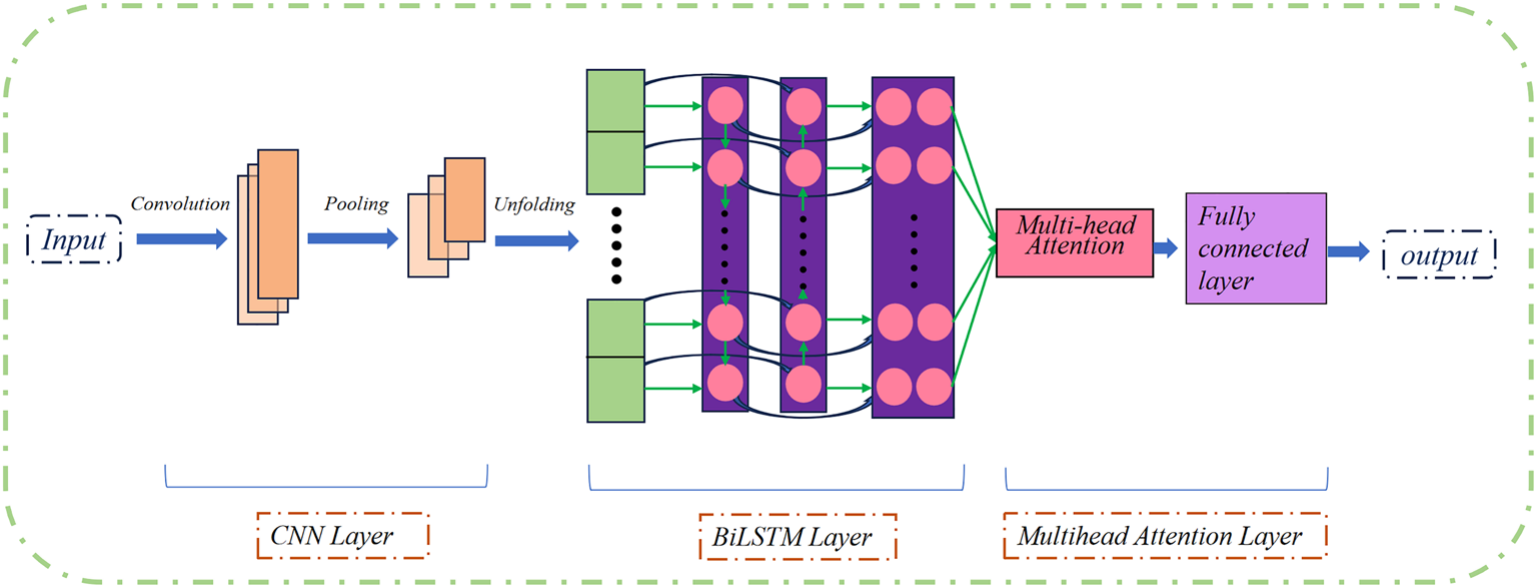
Structure of the CNN-BL-MHA model.

The production data of coalbed methane wells is categorized as time series data. In recent years, LSTM neural networks have been widely employed for predicting time series. However, LSTM networks tend to forget the eigenvalues of longer distances when predicting ultra-long sequences, thereby neglecting the global nature of the sequences. In this paper, we employ a CNN to extract features from the time series data, and a Bi-LSTM network to capture dependencies within the series. However, longer sequences necessitate more step iterations, thereby increasing the computational complexity of the model. Additionally, the iterative processing may hinder the Bi-LSTM’s ability to capture features at longer distances, ultimately affecting prediction accuracy. To address this, we incorporate a multi-head attention mechanism, which enables the model to learn diverse weights and key information within the sequences, effectively capturing the correlations and significance between different locations in the input sequences. Therefore we have constructed the CNN-BL-MHA model, and its architecture is illustrated in Fig. 4.

### Data preprocessing and experimental procedures

#### Data preparation

**Figure 5:**
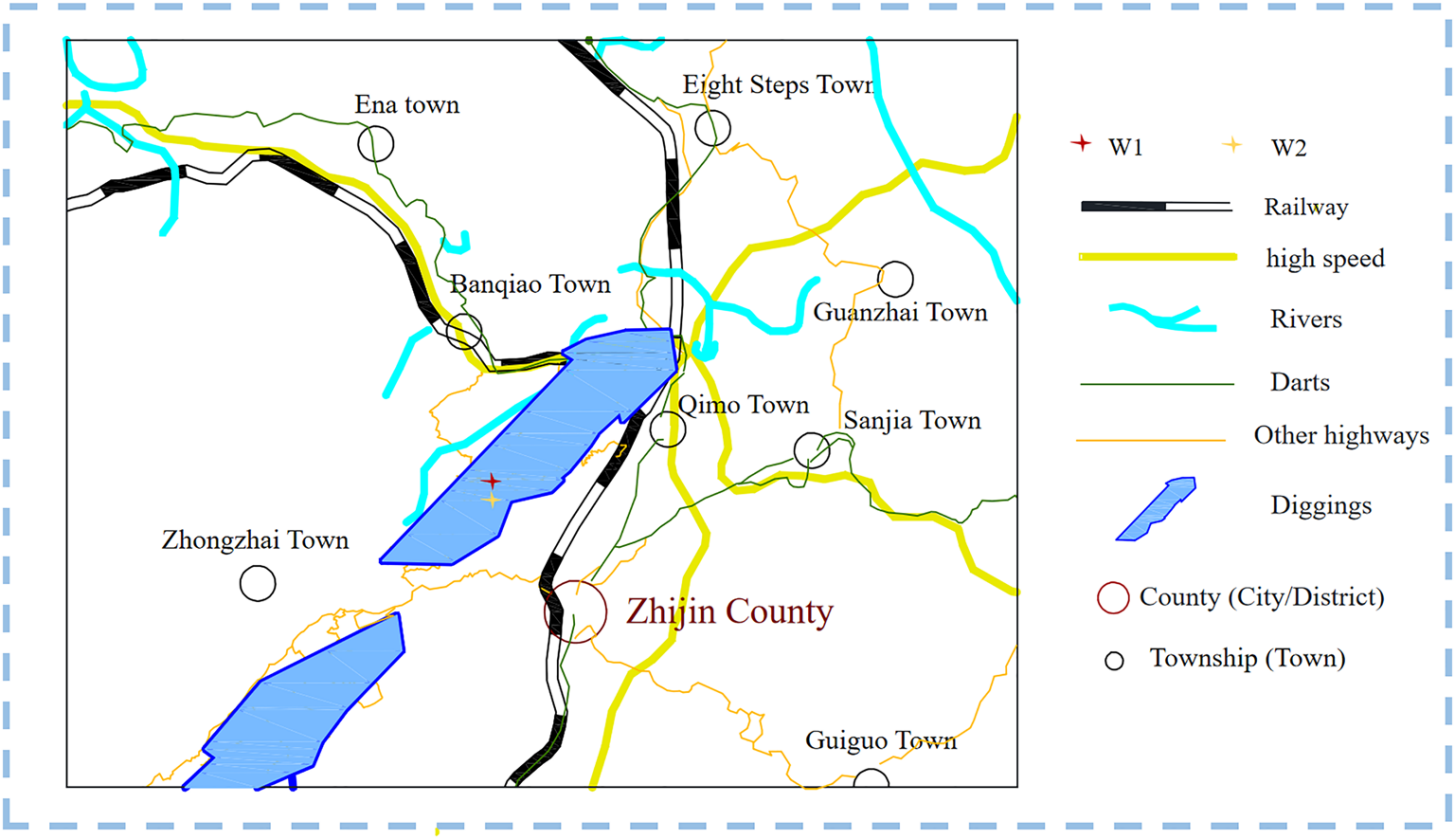
Geographic location map of the mine.

The coal seam under investigation is situated in the Wenjiaba Mining Area, nestled within Zhijin County of Bijie City in Guizhou Province, China. Distinguished by its average permeability of approximately 0.035 mD, it belongs to the category of low-permeability coal seams. Furthermore, boasting an average gas content of 15.86 m3/t, it exemplifies a reservoir rich in gas content. In this study, two comprehensive datasets have been compiled, encompassing the gas production data from two CBM wells in the Wenjiaba mine of Guizhou Province: W1 (Well Number 1) and W2 (Well Number 2), as depicted in Fig. 5.

#### Data preprocessing

During the data collection process, due to various uncontrollable factors such as manual observation errors and sensor accuracy limitations, missing data often occurs in the dataset. Therefore, in the initial stage of data collection, we implemented several preprocessing measures, including outlier elimination and mean imputation. We primarily adopted a time series-based approach for mean imputation, wherein missing values were filled with the mean of the data from the preceding and succeeding week of the missing point. To ensure data integrity and consistency, we subjected the original dataset to a series of refined processing steps, including data cleaning to eliminate anomalies and redundancies, data integration to merge multiple data sources, data transformation for feature engineering, and data reduction to decrease data dimensionality and complexity.

Furthermore, we employed the min–max normalization method, as described in Eq. (9), to standardize the data and ensure consistency and comparability across different dimensions^49^.

9 $$x_{norm} = \frac{{x - x_{\min } }}{{x_{\max } - x_{\min } }}$$

During the data collection process, we primarily focused on gathering daily gas production, casing pressure, bottom hole pressure, and daily water production data from coalbed methane wells. We applied the Principal Component Analysis (PCA) method to explore the correlation between three key factors within this dataset and daily gas production^50^. The results of the PCA analysis revealed a Kaiser–Meyer–Olkin (KMO) measure of sampling adequacy of 0.417, suggesting a relatively low correlation between the factors. Hence, in this experiment, we opted to focus on single-factor modeling for predicting coalbed methane well production.

### Experimental process

**Figure 6:**
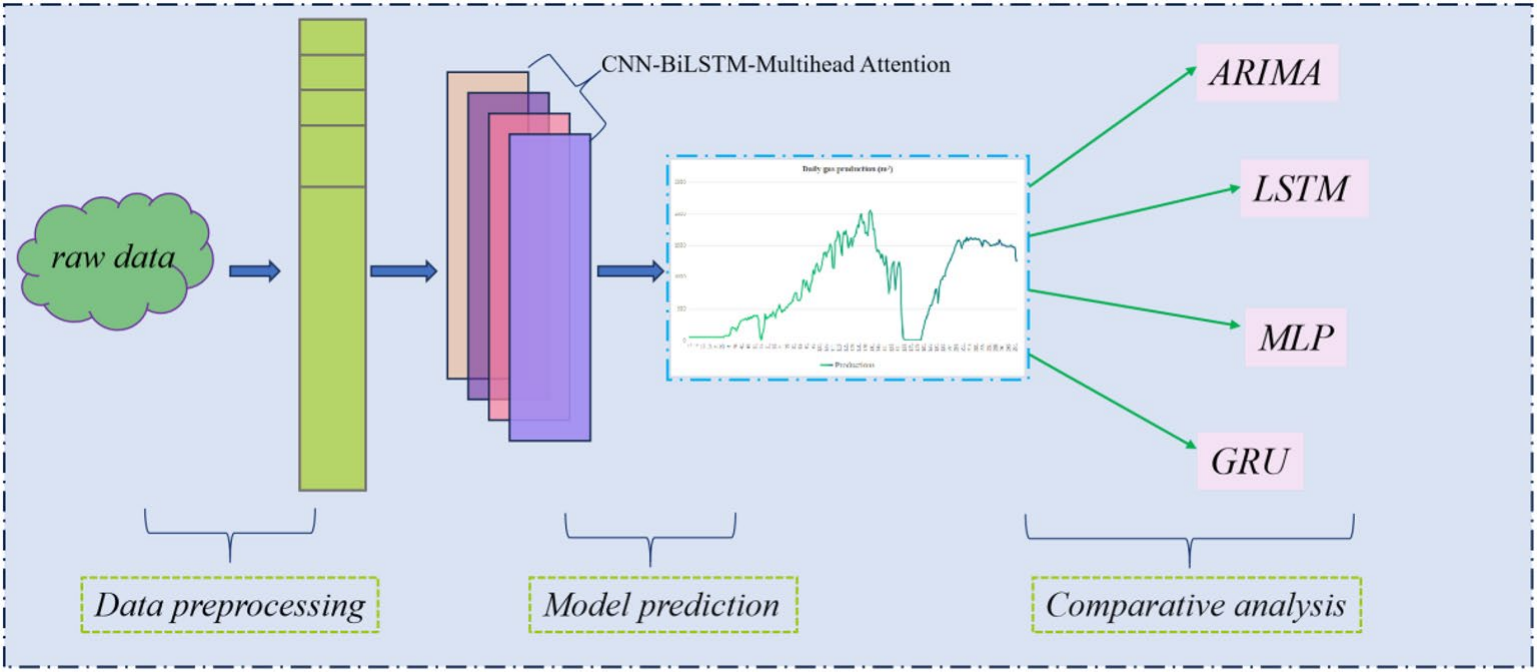
Flow chart of the experiment.

The flowchart depicted in Fig. 6 outlines the methodology adopted in this study. Initially, raw coalbed methane (CBM) production data undergo processing, followed by feature extraction utilizing a CNN. Subsequently, to optimize the production prediction model across multiple levels, we select varying proportions of the total dataset—specifically, 90%, 85%, 80%, and 75%—as the training set. Within each experiment, two-thirds of the data constitute the actual training set, while the remaining one-third is utilized for parameter adjustment. Finally, a comparative analysis is conducted between the predicted results obtained from our approach and those derived from a conventional yield prediction model.

### Comparative test methods

#### ARIMA model

The ARIMA model is a widely utilized time series forecasting approach for yield prediction^51,52^, It incorporates the principles of auto regressive (AR) and moving average (MA) models, along with the application of differencing (I), thereby enhancing its predictive capabilities for time series analysis^53,54^, This can be mathematically represented in Eq. (10).

10 $$~Y_{t} = c + \varphi _{1} Y_{{t - 1}} + \varphi _{2} Y_{{t - 2}} + \cdots + \varphi _{p} Y_{{t - p}} + \theta _{1} \epsilon _{{t - 1}} + \theta _{2} \epsilon _{{t - 2}} + \cdots + \theta _{q} \epsilon _{{t - q}} + \epsilon _{t}$$

where Y_t_ represents the time series data. φ denotes a parameter of the AR (autoregressive) model, characterizing the dependency between the current value and the values at the preceding p time points. θ represents a parameter of the MA (moving average) model, capturing the relationship between the current value and the errors at the prior q time points. ϵ_t_ denotes the error term at time point t.

Where the AR model specifically addresses the autoregressive component of the time series, the differencing (I) operation serves to stabilize non-stationary series and mitigate the impact of uncontrollable external factors, such as weather, thereby enhancing the quality of the time series data for analysis^54^. The MA model is employed to handle the moving average aspect of the time series, accounting for the influence of previous forecasting errors on the present value. The ARIMA model demands a high degree of data quality, necessitates certain levels of smoothness and correlation, and exhibits sensitivity to outliers and exceptional values^54^.

#### LSTM model

**Figure 7:**
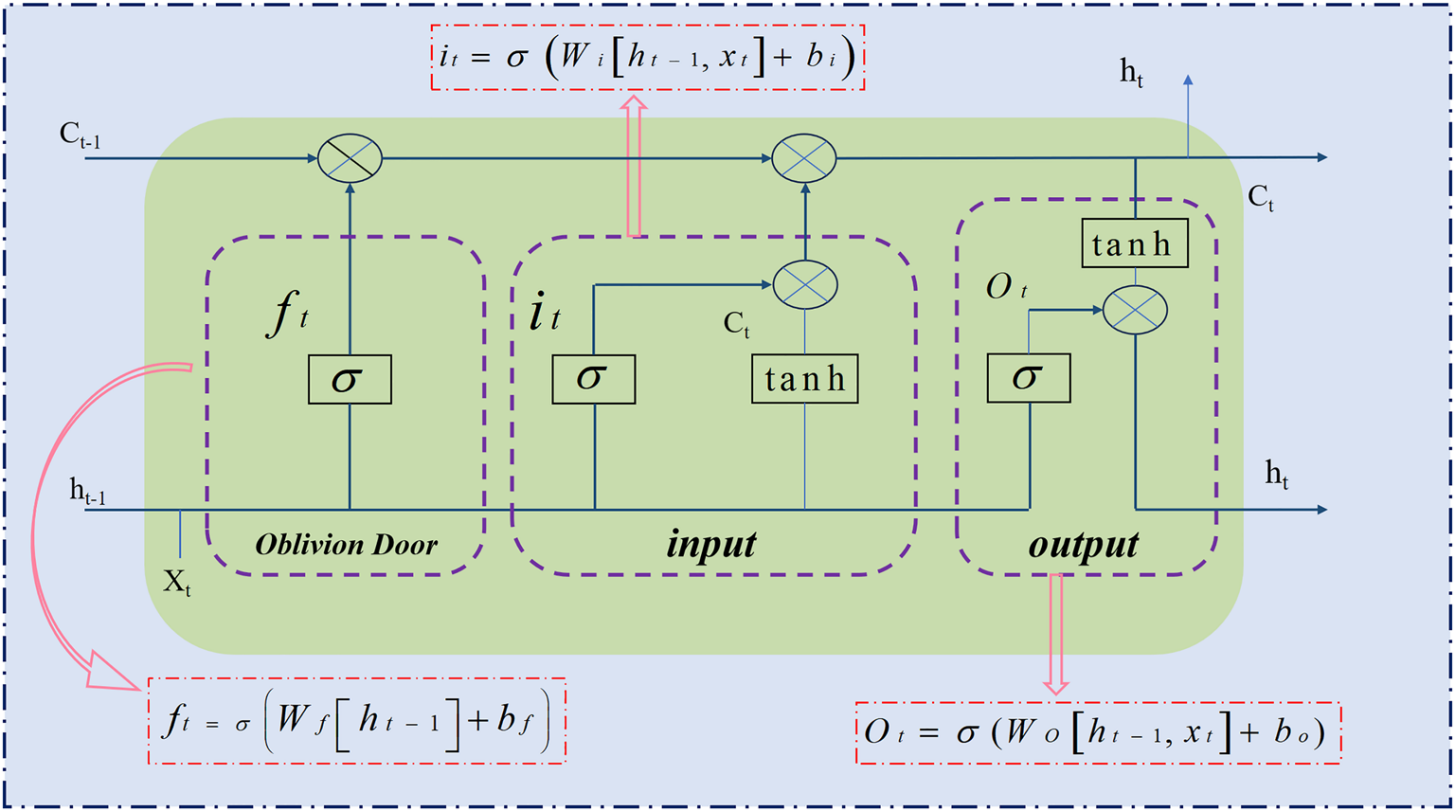
Structure of the LSTM model.

LSTM^23^ represents an advanced refinement of the RNN^55^ architecture, excelling in time series prediction tasks. It primarily utilizes a gating mechanism to effectively retain and discard information, thereby enhancing the capture of long-term dependencies within time-series data^23^. Compared with traditional RNN, As shown in Fig. 7. LSTM regulates the flow of information through the implementation of a gating mechanism. This mechanism primarily encompasses an input gate, a forget gate, and an output gate, allowing for more nuanced control over the retention and dissemination of information.

#### MLP model

**Figure 8:**
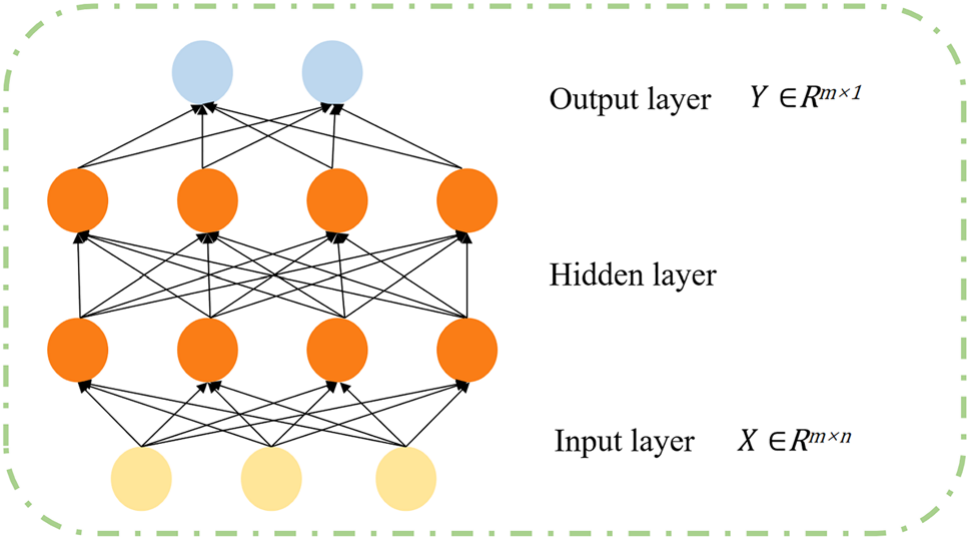
Structure of the MLP model.

As depicted in Fig. 8, the multilayer perceptron (MLP)^56^ represents a prototypical deep learning model, comprised of numerous neurons, where each neuronal layer is fully connected to the subsequent layer. The fundamental structure of the multilayer perceptron (MLP) comprises an input layer, one or more hidden layers, and an output layer^57^. The input layer of the multilayer perceptron (MLP) receives the raw data, while the hidden layer performs a series of nonlinear transformations to map the input data into a higher-dimensional feature space. Ultimately, the output layer transforms the output of the hidden layer into the desired output^56,58^. The prediction algorithm leverages the relationships between feature weights and activation functions, as outlined in Eqs. (6) and (7), to forecast the datasets.

11 $$h_{i}^{l} = f^{l} \left( {\sum\nolimits_{i = 1}^{{U^{l - 1} }} {x_{i}^{l - 1} w_{ij}^{l} } + b^{l} } \right)$$

12 $$f(x) = \frac{1}{{1 + e^{x} }}$$

where *l* represents the total number of layers within the multilayer perceptron (MLP). $f^{l}$ denotes the layer at which the nonlinearity of the MLP is introduced through a mapping function. *b* is the bias vector. *f*(*x*) refers to the sigmoid function, which serves as the activation function.

#### GRU model

The GRU serves as an optimization of RNNs, exhibiting an enhanced memory capacity when dealing with sequential data. In contrast to traditional RNNs, the GRU regulates the flow of information through a gating mechanism, thereby enabling it to more effectively capture long-term dependencies within sequence^59,60^. The Gated Recurrent Unit (GRU) comprises two gating units, namely the Update Gate and the Reset Gate, which distinguish it from the LSTM architecture^59,60^. The update gate within the GRU governs whether the hidden state from the previous timestep should be updated or retained. Meanwhile, the reset gate regulates the degree to which the hidden state of the previous time step is forgotten relative to the input at the current time. By leveraging these two gating units, the GRU can selectively retain or discard information within the sequence, thereby enhancing its capacity to capture long-term dependencies effectively^59,60^.

Where, Update gate:

13 $$Z_{t} = \sigma (W_{z} [h_{t - 1} ,x_{t} ])$$

Reset gate:

14 $$r_{t} = \sigma (W_{r} [h_{t - 1} ,x_{t} ])$$

In the formula, X_t_ represents the input state at the current moment. h_t−1_ represents the hidden state from the previous moment. This hidden state serves as the memory of the neural network, storing information about the data processed by previous nodes. H_t_ denotes the hidden state that will be passed to the next moment. W_r_ represents the weight matrix associated with the reset gate. *σ* is the sigmoid activation function, which transforms the data into a value within the range of 0 to 1. This function helps regulate the flow of information within the GRU.

## Results and discussion

Guizhou, renowned for its distinctive geological landscape, high content of coalbed methane (CBM), exceptional CBM potential, and abundant coal resources in Qianxi and Diandong, stands out as a promising region for CBM development in southern China. A thorough analysis of CBM production across various time series offers valuable insights into its production trends, thereby playing a crucial role in the exploration and development of CBM wells. As previously stated, we categorized the data samples from the two wells into three distinct groups: a training set, a validation set, and a test set. To rigorously assess the prediction accuracy of our model, we experimented with various proportions of the total dataset, specifically 90%, 85%, 80%, and 75%, as the training set. In each experimental iteration, two-thirds of the data served as the actual training set, while the remaining one-third was allocated for parameter tuning, verification, and preventing gradient explosions.

In this section, we employ diverse methodologies and authentic gas production data for a comprehensive comparison. The present approach demonstrates comparable results across both wells, leading us to designate two-thirds of the overall production data as the training set. Furthermore, our study endeavors to predict the data from W1 and W2, contrasting the outcomes with those obtained from other time series prediction models under identical sequence data conditions. This comparative analysis aims to elucidate the strengths and limitations of various methods under uniform circumstances.

### Model training

In the process of model construction, the dataset is first loaded and normalized. Then, a custom CNN-BL-MHA model is defined. Features are extracted first through a one-dimensional CNN layer, which are then passed to a BiLSTM layer for temporal modeling. These features are subsequently enhanced through a multi-head attention layer, and finally, predictions are outputted through global average pooling and a fully connected layer. The parameter settings during training are shown in Table 1:

**Table 1:** Model parameter settings.

Parameter	Input size	hidden_size	output_size	num_heads	kernel_size	Dropout Ratio	activation function	node
value	X.shape [2]	64	1	8	3	0.2	Relu	30

**Figure 9:**
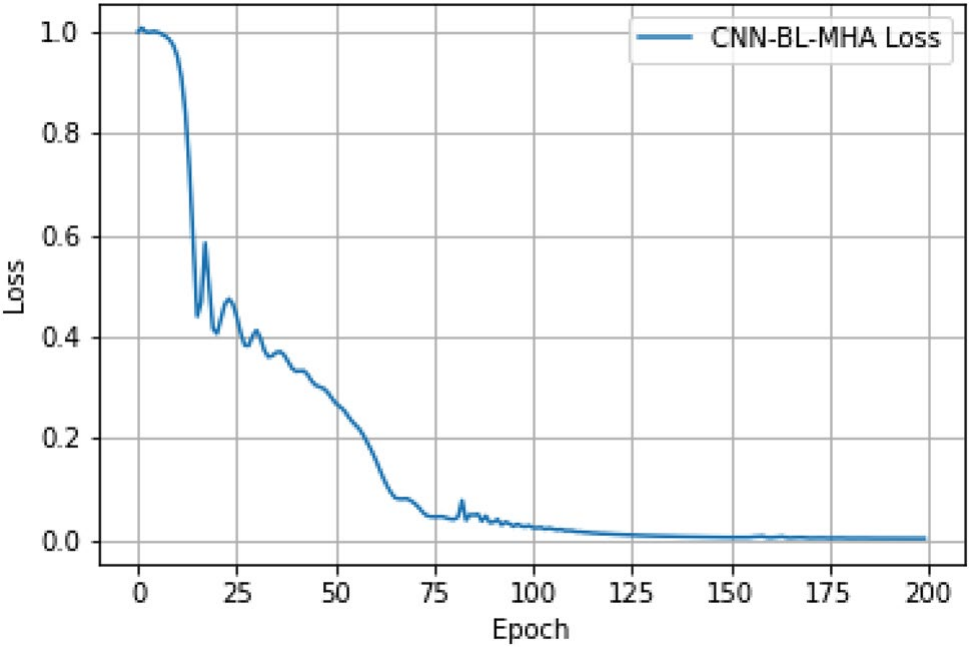
CNN-BL-MHA model loss curve.

And use MSELoss as the loss function, Adam as the optimizer to train the model. During the training process, perform 200 iterations, recording the loss values. Finally, we visualize the loss curve, where the loss function curve decreases from high to low, indicating a reduction in loss and convergence of the model. As shown in Fig. 9.

### Analysis of CNN-BL-MHA model prediction results

**Figure 10:**
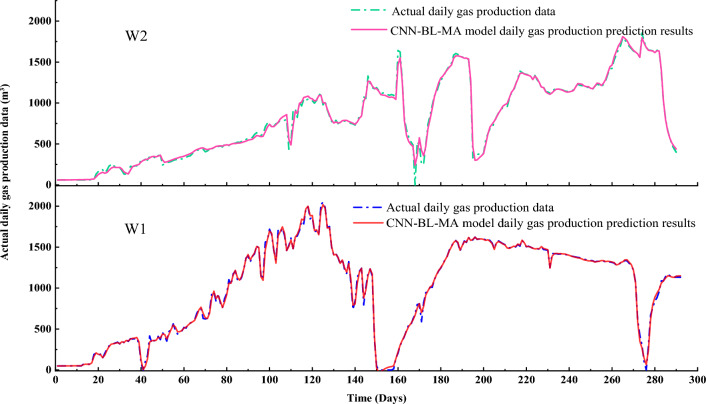
Comparison of actual production data and CNN-BL-MHA model prediction results of two wells.

After establishing the CNN-BL-MHA model using the gas production data from reference wells, when seeking to acquire gas production prediction results for target CBM wells, it suffices to alter the original input data of the model, thus obtaining the desired predictions for the target CBM wells. As depicted in Fig. 10, we contrast the actual production data of wells W1 and W2 with the prediction outcomes generated by the CNN-BL-MHA model. Notably, the coefficient of determination (R2) for W2 is 0.9943, and for W1, it is 0.9983. These values indicate that the experimental model achieves a fitting degree exceeding 98% in predicting the gas production of both wells, thereby demonstrating the model’s high reliability in the prediction process.

To provide a more quantitative assessment of the CNN-BL-MHA model, we calculated and plotted various statistical parameters. Specifically, we utilized four key indicators: Root Mean Square Error (RMSE), Mean Absolute Error (MAE), Mean Square Error (MSE), and Goodness of Fit (R^2^). These parameters serve as robust measures to evaluate the model’s accuracy and determine the level of fluctuation within the yield prediction model. The coefficient of determination (R2) serves as a valuable metric for comparing the performance of our CNN-BL-MHA model against other models trained on identical datasets. The higher the value of R2, the better the model’s fit and predictive capability. MAE represents a loss function that quantifies the aggregate of absolute differences between actual and predicted values. RMSE and MSE are key indicators for a deep understanding of hyperparameter tuning and batch training techniques in deep neural networks. RMSE, specifically, is a widely used evaluation metric, with a range extending from 0 to infinity. Lower values of MAE and RMSE indicate a superior model performance. Table 2 summarizes the combined results of RMSE, MAE, MSE, and R2, providing a comprehensive overview of the model’s effectiveness.

**Table 2:** Different assessment metrics for predicting coalbed methane well production.

Well	RMSE	MAE	MSE	R^2^
W1	23.2517	14.9208	540.6415	0.9983
W2	36.4171	21.9663	1326.2057	0.9943

In the context of predicting coalbed methane well production, these metrics offer valuable insights into the performance of our CNN-BL-MHA model. A high R2 value suggests that the model closely approximates the actual production data, while low MAE, RMSE, and MSE values indicate precise predictions with minimal error. By comprehensively evaluating these metrics, we can gain a comprehensive understanding of the model’s effectiveness in predicting coalbed methane well production. To further demonstrate the superior prediction accuracy of the CNN-BL-MHA model in forecasting coalbed methane well production, we conducted a comparative analysis between the CNN-BL-MHA model and several widely used gas production prediction models. This comprehensive comparison allowed us to quantitatively assess the performance of our model against established baselines, thereby validating its effectiveness in the field of coalbed methane prediction.

### Comparative test result analysis

**Figure 11:**
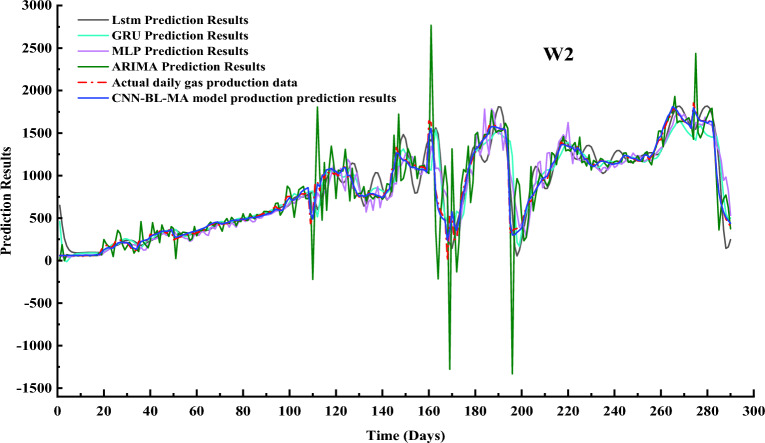
Prediction results of multiple prediction models for gas production from well W2.

**Figure 12:**
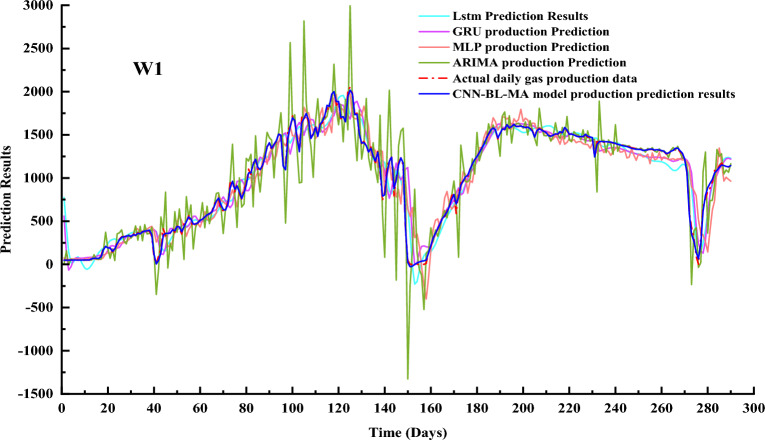
Prediction results of multiple prediction models for gas production from well W1.

The CNN-BL-MHA prediction model establishes a solid foundation for obtaining both actual and predicted historical production values. Through rigorous training, the model is able to accurately determine current predicted values and conduct in-depth studies on future gas production projections. We undertake a comparative analysis of the CNN-BL-MHA prediction model with established techniques such as ARIMA, LSTM, MLP, and GRU. The prediction outcomes of these models are graphically represented as line plots in Figs. 11 and 12, facilitating a visual understanding of their performance. Furthermore, in subsequent experiments, we delve into the strengths and weaknesses of each model by utilizing three error analysis methods: R2, RMSE, and MAE. This comprehensive approach enables us to gain a deeper understanding of the relative merits and limitations of our CNN-BL-MHA model in comparison to other leading prediction models.

As demonstrated in Figs. 11 and 12, utilizing W1 and W2 as illustrative cases, the CNN-BL-MHA prediction model exhibits remarkable nonlinear approximation capabilities. Notably, its predicted gas production outcomes align closely with the actual coalbed methane (CBM) well gas production data, indicating its superiority in accurately forecasting gas production. The CNN-BL-MHA prediction model introduces a multi-head attention mechanism to address the limitations of the Bi-LSTM model in long-term predictions. Notably, the application of the CNN-BL-MHA model to the prediction results of both experimental wells yields significantly more satisfactory outcomes, indicating its enhanced performance and accuracy.

**Figure 13:**
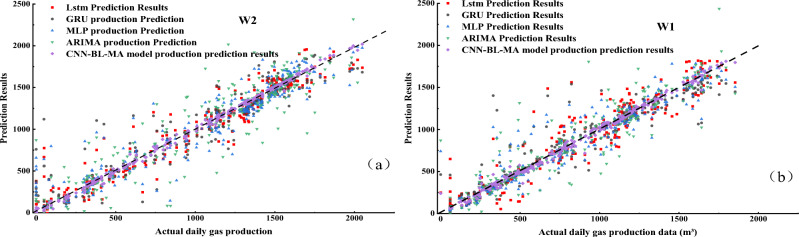
Scattered intersection plot based on multiple gas production prediction method.

To further highlight the strengths of the model, we employ scatter plots to provide a more comprehensive depiction of the prediction results in Figs. 11 and 12. As shown in Fig. 13.

In Fig. 13a,b, the black dashed line represents the optimal result, whereas the blue scatter plot based on the ARIMA model exhibits a notable deviation, indicating its relatively lower prediction accuracy. The prediction accuracies of the MLP and GRU models are comparable and slightly surpass the accuracy of the ARIMA model. Furthermore, the distribution of scatter points in these models is closer to the black dashed line, indicating a closer approximation to the ideal state. This observation also reveals that the accuracy of the ARIMA model decreases notably when the data quality is compromised. In contrast, the prediction accuracies of LSTM are all superior to those mentioned previously. Additionally, the distribution of its red scatter points is more tightly clustered around the black dotted line, which represents the ideal state. This observation also validates the ability of LSTM to effectively memorize dynamic data. The CNN-BL-MHA model exhibits the highest prediction accuracy, with nearly all scatter plots closely clustered around the black dashed line. The results demonstrate that the combination of the Bi-LSTM neural network with the multi-head attention mechanism effectively extracts the dynamic features of gas production data from CBM wells. This model possesses remarkable nonlinear modeling capabilities, enabling more precise predictions of CBM gas production.

Table 3 presents a comparative analysis of the prediction metrics across various models. Based on the test data presented in the table, the CNN-BL-MHA model achieves an outstanding goodness-of-fit, with an R^2^ value exceeding 98%. This finding aligns with the insights derived from the scatter plot, further validating the model’s superior performance. The CNN-BL-MHA model demonstrates exceptional performance in both experimental wells, outperforming other models in terms of prediction accuracy. Specifically, it improves prediction accuracy by 28.17% to 35.83% compared to the ARIMA model, 11.01% to 11.69% compared to the MLP model, 10.24% to 11.73% compared to the GRU model, and 7.25% to 11.99% compared to LSTM. From the aforementioned results, the CNN-BL-MHA prediction model proposed in this study for gas production demonstrates superior performance in experimental wells, garnering favorable feedback. Among the various components, the integration of the multi-head attention mechanism with the bi-directional long and short memory neural network serves as a valuable auxiliary. Given the complexity and high dimensionality of CBM time series data, the multi-head attention mechanism enhances the model’s comprehension of the data, thereby facilitating more accurate predictions of gas production.

**Table 3:** Evaluation indicators for different methods of various forecasting models.

Well	Prediction model	R^2^	RMSE	MAE
W1	ARIMA	0.7789	263.5658	158.5783
	MLP	0.8938	182.7040	126.1305
	GRU	0.9055	172.3649	108.1959
	LSTM	0.9308	147.5546	101.6965
	CNN-BL-MHA	0.9983	23.2517	14.9208
W2	ARIMA	0.7320	250.7766	127.7272
	MLP	0.8962	156.0660	101.2962
	GRU	0.8899	160.7332	87.9603
	LSTM	0.8878	162.2212	108.9963
	CNN-BL-MHA	0.9943	36.4171	21.9663

### Model stability analysis

**Figure 14:**
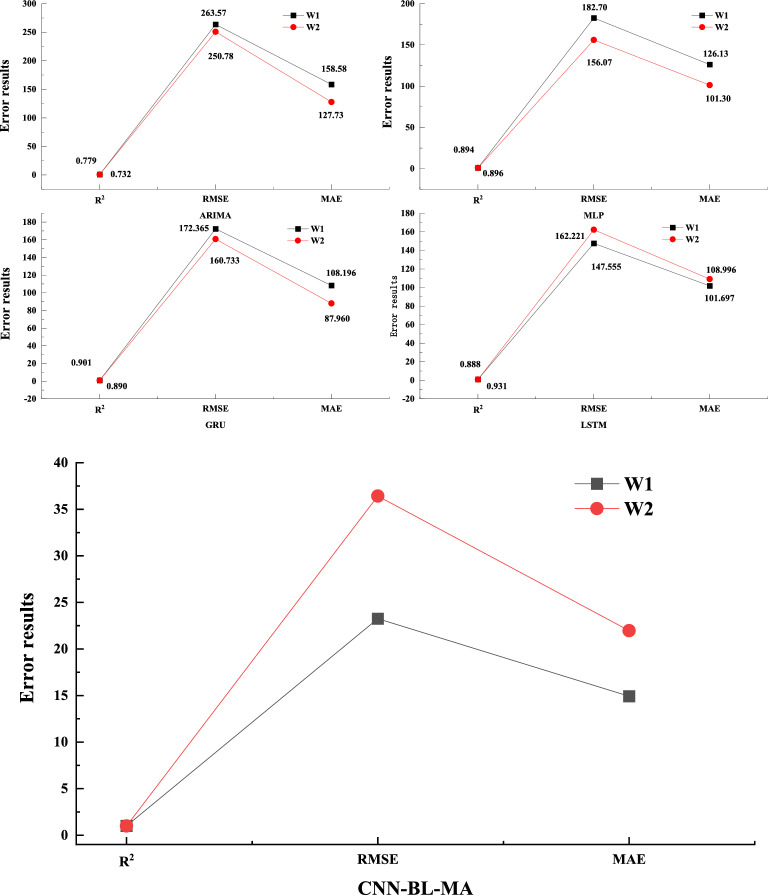
Error analysis of various gas production prediction methods.

Coal bed methane extraction is influenced by numerous uncontrollable factors, resulting in varying degrees of fluctuation in gas production data across different wells. To further corroborate the proposed production prediction model, we have conducted a quantitative analysis of the predictions generated by the same model for different CBM wells, as depicted in Fig. 14. This analysis aims to evaluate the model’s performance and reliability in predicting gas production under diverse conditions.

As depicted in the Fig. 14, the error analysis is graphically represented based on the data presented in Table 3. It is evident that the prediction accuracy of a standalone deep learning yield prediction model exhibits varying degrees of fitting in two distinct experimental wells, with a notable gap observed between them. When comparing the prediction accuracy of various models between well W1 and W2, it is observed that the ARIMA model’s accuracy decreased by approximately 4%, the GRU model’s accuracy decreased by about 2%, and the LSTM model’s accuracy decreased by approximately 4% when predicting well W2. These three models exhibit varying degrees of fluctuation in their fitting capabilities when predicting diverse time series data. Notably, the MLP model and the CNN-BL-MHA model introduced in this study demonstrate less than 1% fluctuation when predicting two distinct sets of time series data. However, the MLP model falls short in prediction accuracy, resulting in a wider discrepancy between its predictions and the actual production data. In summary, the CNN-BL-MHA yield prediction model proposed in this study not only exhibits high accuracy but also demonstrates robust stability and broad adaptability.

### General discussion

The ARIMA model necessitates stationary data to enhance forecasting accuracy. Based on this, MLP amplifies its nonlinear modeling capabilities via multiple hidden layers, thereby enabling better handling of time series data and capturing complex, nonlinear relationships. Nonetheless, MLP risks overfitting with intricate datasets and underfitting when data is scarce. GRU, a derivative of RNN, incorporates two gated units, proving more proficient in processing time series data than MLP. However, its performance may suffer with intricate time series, and its sensitivity to input order can undermine stability. LSTM, an advancement of RNN, boasts three gated units, offering a more intricate structure than GRU but greater flexibility, permitting the disregard of inconsequential information and bolstering modeling prowess. Yet, its elaborate structure may hinder its utility in extensive computations, potentially overlooking distant data traits and diminishing forecasting precision.

To overcome the constraints of these models, we introduce a cutting-edge forecasting model that integrates CNN, Bidirectional Long Short-Term Memory Neural Networks, and a Multi-Head Attention Mechanism. This comprehensive approach targets enhanced time series data processing. The new model precisely captures temporal traits and automatically learns sequence dependencies, elevating forecasting accuracy significantly. Its complexity, however, might impede its application in swift-response scenarios like real-time gas production forecasting. Moreover, the model’s stability and generalization performance across diverse geological conditions await further validation. Nonetheless, this innovative approach presents fresh insights and solutions for time series data processing.

## Conclusions

In this paper, we introduce the CNN-BL-MHA model for predicting coalbed methane well production. This model utilizes CNN to preprocess data efficiently and integrates Bi-LSTM with a multi-head attention mechanism to develop a precise and robust coalbed methane well production prediction framework. Through meticulous discussion and analysis of field data, the following conclusions have been derived:

1. The CBM well production prediction model, which incorporates a multi-head attention mechanism, is capable of extracting the dynamic features of CBM well production in a more comprehensive manner. As a result, the model achieves a prediction accuracy of over 98% for future production, with specific accuracy rates of R_W1_^2^ = 99.83% and R_W2_^2^ = 99.43%. This significant enhancement in prediction accuracy offers valuable technical guidance for the future development of the CBM industry.
2. The CNN-BL-MHA gas production prediction model is comprehensively compared to various standalone deep learning models, including ARIMA, GRU, MLP, and LSTM. In this study, the proposed CBM production prediction model demonstrates superior performance in two experimental wells, achieving a significant improvement in prediction accuracy of approximately 7.25% to 35% within the same dataset. The CNN-BL-MHA model’s integration of the multi-head attention mechanism confers enhanced flexibility and adaptability when dealing with production data from diverse wells. Consequently, the CNN-BL-MHA gas production prediction model effectively realizes accurate predictions of CBM production.
3. Artificial intelligence has exhibited considerable potential and benefits across numerous fields, yet deep learning encounters numerous challenges in the realm of CBM production prediction. Key among these challenges are the variable quality and completeness of data, which can significantly influence the model’s prediction accuracy. Additionally, the generalization capabilities of the model require further enhancement. To address these issues, future research and studies should prioritize interdisciplinary collaboration, integrating artificial intelligence with geology, geophysics, and other pertinent disciplines. Such an approach will facilitate the optimization of the CBM production prediction model, leading to more accurate and reliable predictions.

## Data Availability

All data used during this research are available from the corresponding author by reasonable request.
